# Prevalence and molecular characterizations of enterovirus D68 among children with acute respiratory infection in China between 2012 and 2014

**DOI:** 10.1038/srep16639

**Published:** 2015-11-16

**Authors:** Qiuyan Xiao, Luo Ren, Shouyan Zheng, Lili Wang, Xiaohong Xie, Yu Deng, Yao Zhao, Xiaodong Zhao, Zhengxiu Luo, Zhou Fu, Ailong Huang, Enmei Liu

**Affiliations:** 1 Ministry of Education Key Laboratory of Child Development and Disorders, Key Laboratory of Pediatrics in Chongqing, Chongqing International Science and Technology Cooperation Center for Child Development and Disorders, Chongqing, 400014, China; 2 Department of Respiratory Medicine, Children’s Hospital of Chongqing Medical University, Chongqing, 400014, China; 3 Key Laboratory of Molecular Biology of Infectious Diseases, Ministry of Education, Chongqing Medical University, Chongqing, 400014, China

## Abstract

EV-D68 is associated with respiratory tract infections (RTIs). Since its first isolation, EV-D68 has been detected sporadically. However, the US and Canada have experienced outbreaks of EV-D68 infections between August and December 2014. This study aimed to investigate the molecular epidemiology and clinical characteristics of EV-D68 in Chongqing, Southwestern China. From January 2012 to November 2014, 1876 nasopharyngeal aspirate specimens (NPAs) were collected from hospitalized children with RTIs. Among the 1876 NPAs, EV-D68 was detected in 19 samples (1.0%, 19/1876). Of these, 13 samples were detected in September and October 2014 (9.8%, 13/132). Phylogenetic analysis showed that all 13 strains detected in the 2014 Chongqing had high homology with the main strains of the 2014 US outbreak. Among the children with EV-D68 infection, 13 (68%) had a history of recurrent wheezing. A total of 13 children had a discharge diagnosis of asthma. Of these, 11 children were diagnosed with acute asthma exacerbation. EV-D68 was the predominant pathogen that evoked asthma exacerbation in September and October 2014. In conclusion, our results found that a history of recurrent wheezing may be a risk factor for the detection of EV-D68 and viral-induced asthma exacerbation may be a clinical feature of EV-D68 infection.

Enterovirus D68 (EV-D68) belongs to human enterovirus species D of the genus *Enterovirus* within the family *Picornaviridae*. EV-D68 was originally isolated from four children with pneumonia and bronchiolitis in California, United States (US), in 1962^1^. In contrast to other enteroviruses, EV-D68 is associated with respiratory tract infections (RTIs) because EV-D68 is an acid-sensitive virus that is biologically more similar to human rhinoviruses^2^. Since its first isolation, EV-D68 has been detected sporadically, and only 26 isolates were identified in the US enterovirus surveillance between 1970 and 2005^3^. However, between 2008 and 2010, increased reports of worldwide circulation of EV-D68 have been published in Asia, Europe, Africa, and the US^4^,^5^,^6^. Besides RTIs, EV-D68 infection has been associated with rare cases of central nervous system (CNS) disease^3^,^7^.

The US and Canada have experienced widespread outbreaks of EV-D68 infections associated with severe respiratory disease since mid-August 2014. Underlying conditions such as asthma or wheezing have been reported in approximately 70% to 80% of EV-D68 cases^8^. More critically, Messacar K *et al.* found that the outbreak of EV-D68 was geographically and temporally associated with acute flaccid paralysis (AFP) and cranial nerve dysfunction in children^9^. There was a case report of AFP following EV-D68 infection in Europe in which EV-D68 was isolated from nasopharyngeal aspirate, stool and bronchoalveolar fluid specimens of the child^10^.

In a previous hospital-based viral surveillance study of RTI cases in Chongqing, China, from 2009 to 2012, the detection rate of EV-D68 was 0.4% (7/1565)^11^. However, the specific clinical characteristics and gene structural features of EV-D68 in China remain unclear. Therefore, the current study aimed to investigate the molecular epidemiology and clinical characteristics of EV-D68 in Chongqing, China.

## Results

A total of 1876 NPAs were collected from children with RTIs between January 2012 and November 2014 (559 from 2012, 631 from 2013, 686 from 2014). Children enrolled in this study were of ages ranging from 1 month to 16 years and 11 months (median, 9 months). The overall male-to-female ratio was 1238: 638, with a 1.9 bias towards males. Among all samples, 1476 (78.7%) were positive for at least one viral agent. RSV was the most common viral agent (detected in 572 children [30.5%]), followed by HRV (473 children [25.2%]), PIV (384 children [20.5%]), HBoV (258 children [13.8%]), IV (190 children [10.1%]), ADV (110 children [5.9%]), HMPV (44 children [2.3%]), HCoV (27 children [1.4%]) and HEV (26 children [1.4%]). Among the 26 HEV positive samples, 19 were positive for EV-D68 (1.0%, 19/1876, Fig. 1). The frequency of EV-D68 detection was 0.4% (6/1681) from January 2012 through August 2014 and 9.8% (13/132) from September through October 2014. Moreover, the detection rate of EV-D68 in September-October 2014 was higher than in September-October 2012 (9.8%, 13/132 vs. 0%, 0/42, *P* = 0.04) and in September-October 2013 (9.8%, 13/132 vs. 2.4%, 3/127, *P* = 0.012).

**Figure 2:**
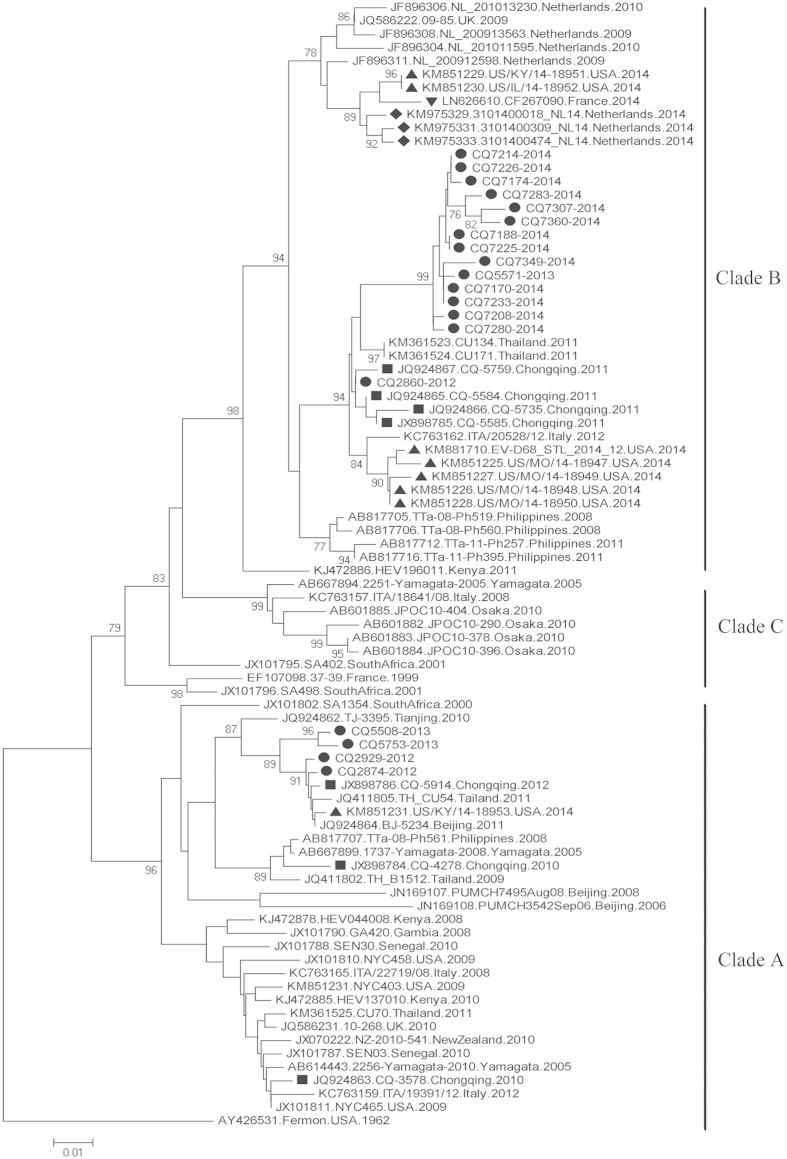
Phylogenetic tree of selected EV-D68 strains based on the nucleotide sequence of VP1 genome regions. The 369-bp fragments, which correspond to nt 2518-2886 of the EV-D68 prototype strain (GenBank accession no. AY426531), were used to construct the phylogenetic tree. ● Strains detected in this study. ■ EV-D68 previously detected in Chongqing from 2009–2012. ▲ Sequences from the US in 2014. ♦ Sequences from the Netherlands in 2014. ▼ Sequences from France in 2014.

**Figure 3:**
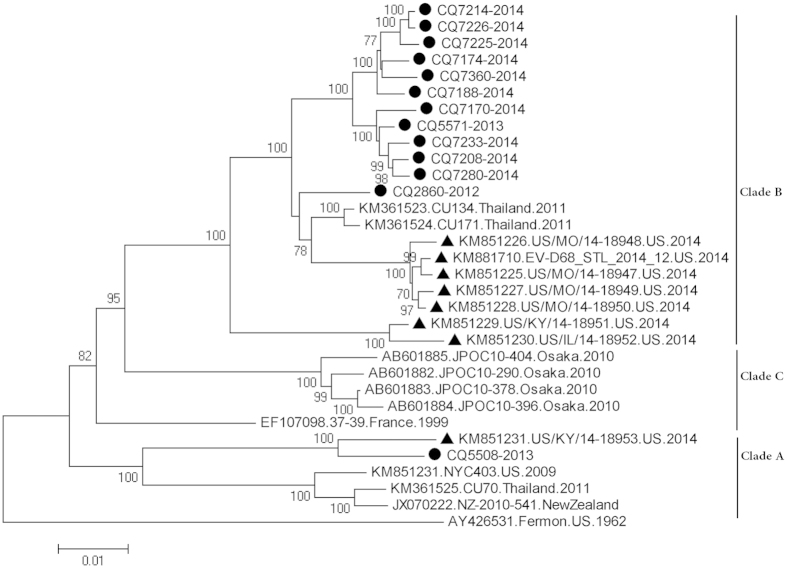
Phylogenetic tree of selected EV-D68 strains based on the nucleotide sequence of 5′UTR to 3D genome regions. The 7059-bp fragments, which correspond to nt 145-7203 of the EV-D68 prototype strain (GenBank accession no. AY426531), were used to construct the phylogenetic tree. ● Strains detected in this study ▲ Sequences from the US in 2014.

Sequence analysis of VP1 revealed that the 19 strains in Chongqing could be grouped into two clades (clade A and clade B) that were the main epidemic genotypes in Chongqing from 2009 to 2012 (Fig. 2)^11^. Thirteen strains of 2014 Chongqing detected, seven strains from the 2014 US outbreak, and most strains from the 2014 Netherlands detected were grouped into clade B. However, only two Chongqing strains from 2012 and 2013 could be grouped into clade B. The remaining four Chongqing strains were grouped into clade A (two in 2012, and two in 2013). Unfortunately, only thirteen Chongqing strains were nearly full-length nucleotide sequences, including ten strains from 2014, two strains from 2013, and one strain from 2012. Sequence analysis of nearly full-length nucleotide sequences found that the 13 clinical isolates detected in the current study had nucleotide sequence similarities of 89.9–99.8% with each other and similarities of 87.8–88.2% with the Fermon strain. Strains of clade B demonstrated similarities ranging between 94.3–99.8% (nucleotides). A phylogenetic tree constructed with nearly full-length nucleotide sequences revealed that the division of EV-D68 was consistent with Fig. 2 (Fig. 3).

**Figure 4:**
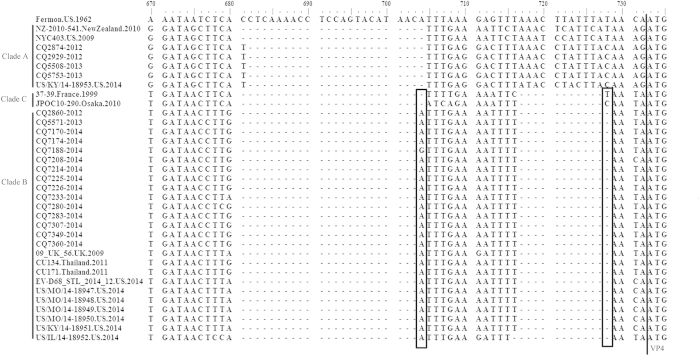
Nucleotide regions of 5′untranslated regions of EV-D68 showed the deletion blocks preceding VP4. The difference in deletions (position 704 and 728) between clade B and clade C are indicated by boxes.

Kaida *et al.* first reported that EV-D68 Osaka strains have two deletions at nt 681–704 and 717–727 in contrast to the Fermon strain in the 5′UTR^12^. These EV-D68 Osaka strains were grouped into clade C according to phylogenetic analysis (Fig. 2). In the current analysis, fifteen Chongqing strains and seven strains from the 2014 US outbreak that were grouped into clade B also had two blocks of deletions at nt 681–703 and 717–728 in contrast with the Fermon strain (Fig. 4), which differed from clade C at nt 704 and 728. Tokarz *et al.* found that clade A only had a deletion at nt 681–704^4^. In the current analysis, four Chongqing strains (CQ2874, CQ2929, CQ5508, and CQ5753) and one strain from the 2014 US outbreak (US/KY/14-18953) that were grouped in clade A had a deletion at nt 682–704, which contrasted with the Fermon strain by a nucleic acid substitution at position 681 (Fig. 4).

The complete structural viral protein (VP4-VP1) amino acid sequences were also analyzed. Clade B had clearly different amino acid signatures relative to the prototype Fermon strain and had different signatures relative to clades A and C (Figure S1). However, all clade B amino acid residue substitutions have been reported in previous studies^4^,^5^,^6^,^13^,^14^,^15^.

The clinical and epidemiological characteristics of 19 children positive for EV-D68 are shown in Table 1 and 2. The children with EV-D68 ranged in age from 1 month to 12 years and 8 months (median, 32 months). The male-to-female ratio was 10:9. The most common signs and symptoms exhibited by the children were cough (100%) and wheezing (90%), with only five (26%) children having fever. Thirteen (68%) children had a history of recurrent wheezing (Table 2). Thirteen children had a discharge diagnosis of asthma, in which twelve children dually diagnosed with pneumonia. Among these thirteen children, eleven were diagnosed with acute asthma exacerbation. Moreover, three children were diagnosed with pneumonia, one child was diagnosed with bronchiolitis, one child was diagnosed with *Mycoplasma* pneumonia, and one child was diagnosed with bronchitis (Table 1). EV-D68 was detected as a sole pathogen in 8/19 cases (with the exception of other common respiratory viruses, bacteria, and *Mycoplasma*). Children diagnosed with asthma were more commonly associated with cases of EV-D68 single detection than co-detection (100% vs. 45%, *P* = 0.018, Table 2). The clinical isolates of EV-D68 in 2014 were all in clade B according to phylogenetic analysis. Comparisons were made between clades B and A (Table 2). Children infected with clade B viruses were significantly older than those infected with clade A viruses (median of 43 months old vs. 4.5 months old, *P* = 0.001). The duration of hospitalization was shorter for clade B-infected children than for clade A-infected children (median of 5 days vs. 8.5 days, *P* = 0.027). Diagnoses of asthma and acute asthma exacerbation were more common among children infected with clade B viruses than those with clade A (87% vs. 0%, *P* = 0.004; 73% vs. 0%, *P* = 0.018). Moreover, among the thirteen clinical isolates of EV-D68 in 2014, eleven cases were among children diagnosed with acute asthma exacerbation. Of these, five and six cases were among children diagnosed with severe and moderate acute asthma exacerbations, respectively (Table 1).

Among the 1876 NPAs samples, 75 were obtained from children admitted to the hospital with acute asthma exacerbation. The pathogen spectrum among the children with acute asthma exacerbation is shown in Table 3 with HRV and RSV predominating. HRV and RSV were the predominant pathogens associated with acute asthma exacerbation in September and October of 2012 and 2013. EV-D68 was the predominant pathogen in September and October of 2014 following the decline of HRV.

## Discussion

Combined with the results of the previous study in Chongqing, thirteen cases of EV-D68 were detected during the period 2009–2013: two in 2010, four in 2011, four in 2012, and three in 2013^11^. EV-D68 infection was substantially higher in September and October 2014 than in previous years.

Recent studies have shown that EV-D68 infection presents from mild respiratory illness to severe acute lower respiratory tract infection and even death^3^,^8^,^11^,^12^,^13^,^14^,^15^,^16^,^17^,^18^. Additionally, increasing evidence supports the relationship between EV-D68 infection and AFP^3^,^7^,^9^,^10^. Hasegawa *et al.* found a strong association between EV-D68 infection and exacerbation of asthma^19^. In the 2014 EV-D68 outbreak in the US, the percentage of children who had a history of asthma or wheezing in Kansas City and Chicago was 68% and 73%, respectively, and most of these children were admitted to the pediatric intensive care unit^8^. At the St. Louis Children’s Hospital in St. Louis, Missouri, EV-D68 was detected in seven of ten samples from patients with severe disease^20^. In our study, most children infected with EV-D68 had a history of recurrent wheezing or asthma and a diagnosis of acute asthma exacerbation. In EV-D68 single detection, the phenomenon was more obvious. Moreover, EV-D68 became the predominant pathogen associated with asthma exacerbation in September and October of 2014. Our study found that a history of recurrent wheezing or asthma may be a risk factor for the detection of EV-D68 and viral-induced acute asthma exacerbation may be a clinical feature of EV-D68 infection in Chongqing area.

The current study found that clade B viruses had a stronger association with asthma exacerbation than clade A, but children infected with clade A viruses were too young to be diagnosed with asthma^21^. Therefore, the difference between clade B- and clade A-induced asthma exacerbations could not be inferred. In addition, the duration of hospitalization was longer in clade A-infected children. However, the number of cases was too small to estimate differences in disease severity between clade B and clade A. Further studies are thus needed to explore additional relationships between different clades.

The 5′UTR contains the internal ribosome entry site (IRES) responsible for cap-independent initiation of translation as well as other secondary structural elements responsible for genome replication^22^. Studies have found that the enterovirus IRES is associated with translational efficiency and virulence of the enterovirus^4^,^23^,^24^. The 5′UTR spacer region of EV-D68 ranged from the end of the IRES and the polyprotein open reading frame (ORF)^4^. The role of the spacer region in viral fitness remains unclear^4^. Deletions in the spacer region might be correlated with the worldwide increase in EV-D68^4^,^12^. Our study showed different deletions among clades A, B and C. Clade A only had one deletion (nt 682–704)^4^, clade C had two deletions (nt 681–704 and 717–727)^12^, and clade B had two deletions (nt 681–703 and 717–728)^5^. The 5′UTR was considered a conserved region but the spacer region likely had more mutations. Therefore, more sequences would need to be collected to explore the nucleic acid differences of 5′UTR sequences among different clades. More studies are needed to expound the relationship between the deletions within the 5′UTR of EV-D68 and efficiency of translation, or virulence.

The VP1 contains a serotype specific neutralization site, the BC and DE loops, associated with viral antigenicity^6^. Previous studies found some distinctively different amino acid substitutions among the three clades, particularly in the BC and DE loops of the VP1 protein^4^,^5^,^6^,^13^,^14^,^15^. Such changes might modify the immune response and help to explain the biological basis for the increase in EV-D68 incidence^4^,^13^. However, an association between the substitutions and the severity of infection has not been established^25^. Phylogenetic analysis of VP1 sequences suggested that the major EV-D68 epidemic clade varied across time. The epidemic clades of EV-D68 in the Philippines (2008), New York (2009), Japan (2010), and the Netherlands (2010) were clade B, clade A, clade C and clade A, respectively^12^,^16^,^17^,^26^. The predominant clade might shift from clades A and C to clade B, and clade B might expand worldwide. Although the main 2014 EV-D68 epidemic clade in Chongqing, the US, and the Netherlands was clade B, it was different from the epidemic cluster observed in the Philippines in 2008, and no prior increase was reported. Moreover, the EV-D68 strains in certain areas had regional features as evidenced by the fact that the EV-D68 epidemic strains in Chongqing and US belonged to a different cluster than the Netherlands strains. This phenomenon may be linked to the rapid evolution of enteroviruses^27^.

Our study has several limitations. First, as this study was limited to inpatients, we could not analyze the clinical characteristics in outpatients and asymptomatic children. Viral surveillance was performed in a tertiary referral hospital where diseases seen were often more severe, thus more EV-D68 might have been detected. Second, because we only collected the NPAs of children with RTIs, no children positive for EV-D68 in our study had muscle weakness or paralysis. We could not find any relationship between AFP and EV-D68.

In summary, we found that clade B of EV-D68 increased in September and October 2014 in Chongqing, China. A history of recurrent wheezing or asthma appeared to be a risk factor for the detection of EV-D68 and viral-induced acute asthma exacerbation might be a clinical feature of EV-D68 infection. Our study provides additional epidemiological and clinical data on EV-D68 infection in China and a resource of genome sequences for genomic comparison of EV-D68. At present, EV-D68 is not included in the viral surveillance of ARTIs in China, but it has emerged as a considerable global public health threat. Additional surveillance of EV-D68 is needed.

## Methods

### Ethics statement

This study was approved by the Ethics Committee of the Children’s Hospital of Chongqing Medical University. The study was conducted in compliance with the principles of the Declaration of Helsinki. Informed consent was obtained from each parent or guardian prior to enrollment.

### Patients and sample collection

The study was conducted at the Department of Respiratory Medicine at the Children’s Hospital of Chongqing Medical University (CHCMU) in Chongqing, China. Chongqing is located in a subtropical region, and CHCMU is the largest children’s hospital in southeastern China. Nasopharyngeal aspirates (NPAs) were collected from children with RTIs^11^. A standardized questionnaire was designed to obtain the patients’ information from medical records, including demographic characteristics, underlying medical conditions, symptoms and signs, laboratory tests, chest radiograph results, hospital course, and discharge diagnoses.

### Clinical assessments

We defined four clinical variables related to wheezing: (1) a history of asthma, which was determined by asking the parents whether their child had ever been given a definitive diagnosis of asthma by clinicians or by documentation of a previous diagnosis of asthma in the medical record; (2) a history of recurrent wheezing, which was determined by asking the parents whether their child had ever had the symptom of wheezing at least three times; (3) a discharge diagnosis of asthma, which was defined as any discharge diagnosis of asthma following the guidelines of the Global Initiative for Asthma (GINA);^21^ and (4) hospitalization for acute asthma exacerbation using criteria in accordance with previous and current published international guidelines^28^. The degrees of acute asthma exacerbations were divided into mild, moderate and severe^28^.

### Molecular analysis

Viral DNA and RNA were extracted from 200-μL aliquots of the NPA samples by a QIAampMinElute Virus Spin kit (Qiagen, Hilden, Germany). RNA was applied as the template for cDNA synthesis using the SuperScript III First-Strand Synthesis System (Invitrogen, California, USA). All experimental methods were carried out according to the manufacturers’ instructions.

All samples were screened for influenza viruses (IVA-C), human parainfluenza viruses (PIV1-4), respiratory syncytial virus (RSV-A and RSV-B), human coronaviruses (HCoV-229E and HCoV-OC43), human metapneumovirus (HMPV), adenovirus (ADV) and human bocavirus (HBoV) using molecular methods, as described previously^29^,^30^. A primer pair targeting 400 bp of the 5′untranslated regions (5′UTR) of the human enterovirus (HEV) and human rhinovirus (HRV) genome was used to distinguish between HEV and HRV^16^. To determine EV-D68 sequences, we synthesized cDNA using specific primer pairs (Table 4)^13^,^17^. The sequenced regions were all structural viral proteins (VP4-VP1), partial 5′UTR regions, and nonstructural viral proteins (2A-3D). PCR was performed using a TaKaRa *Ex* Taq PCR kit (Takara Biotechnology, Dalian, China), and all PCR products were sequenced by Shanghai Majorbio Bio-Pharm Technology.

### Detection of bacteria and yeasts

Qualitative and semiquantitative cultures for bacteria and yeasts were performed using standard microbiological methods. For all samples, macroscopically distinct colonies were isolated from pure culture, and standard methods for identification, typing and establishing antibiotic sensitivity patterns were used^31^.

### Sequence analysis

Sequence data for each clinical strain were formatted and assembled by the Seqman program of DNASTAR Software (v5.0). The sequences of EV-D68 strains that were newly obtained in Chongqing have been deposited in GenBank under the accession numbers KT803582–KT803606. To study the distribution and diversity of EV-D68 identified in this study compared to other EV-D68 strains, multiple sequence alignments were made using the ClustalW function of the BioEdit program (v7.0.9). Phylogenetic trees were constructed using the MEGA neighbor-joining algorithm (5.05). The statistical significances of the tree topologies were tested via bootstrapping (1000 replicates). Only bootstrap values >70% are shown in each tree. A phylogenetic tree that was constructed with 369 bp of VP1 gene nucleotide sequences showed that modern EV-D68 strains were divided into three primary clades (A, B and C) according to the classification previously described by Tokarz (Fig. 2)^4^. Nearly full-length nucleotide sequences that correspond to nt 145–7203 of the EV-D68 prototype strain (GenBank accession no. AY426531) were obtained from 13 strains of EV-D68 in the current study. A phylogenetic tree was constructed based on this region of nucleotide sequences with the 13 strains from Chongqing (Ten strains from 2014, two strains from 2013, and one strain from 2012), seven strains from the 2014 US outbreak and available EV-D68 sequences that were long enough (Fig. 3).

### Statistical analysis

Data were analyzed using the SPSS 17.0 software package. Categorical variables were compared using chi-square or Fisher’s exact tests, and continuous variables were compared using the nonparametric Mann-Whitney U-test. Two-sided *P*-values of <0.05 were considered statistically significant.

## Additional Information

**How to cite this article**: Xiao, Q. *et al.* Prevalence and molecular characterizations of enterovirus D68 among children with acute respiratory infection in China between 2012 and 2014. *Sci. Rep.* **5**, 16639; doi: 10.1038/srep16639 (2015).

## Supplementary Material

Supplementary Information

## Figures and Tables

**Figure 1:**
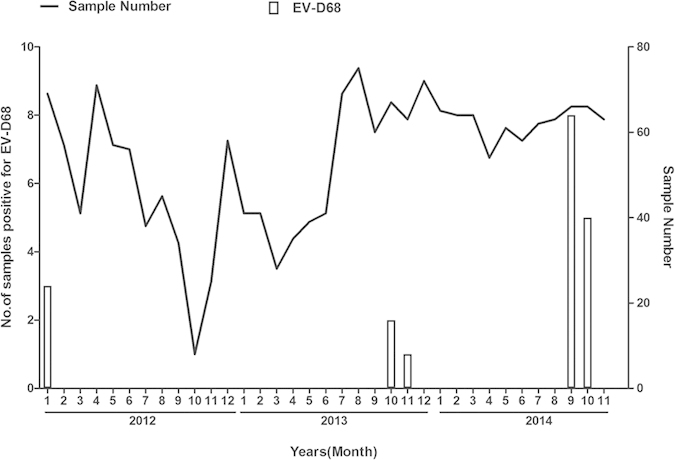
Monthly distribution of EV-D68 in Chongqing, China, January 2012 to November 2014. Bars indicate the number of specimens that were positive for EV-D68 and the line indicates the number of specimens tested.

**Table 1:** Characteristics of patients with EV-D68 infection between 2012 and 2014, Chongqing, China.

Patient no.	Clade	Underlying medical conditions	Age/sex	Disease onset date	Signs/symptoms	diagnosis	Co-detected pathogens
CQ2860	B	Asthma	1y4mo/M	Jan 2012	Cough and wheezing	Pneumonia, asthma	IV-A
CQ2874	A	Recurrent wheezing	0y7mo/F	Jan 2012	Cough and wheezing	Pneumonia	IV-A *Streptococcus pneumoniae, Haemophilus influenzae, Moraxella catarrhalis*
CQ2929	A	No	0y7mo/M	Jan 2012	Fever, cough, diarrhea, and wheezing	Bronchiolitis	RSV-B
CQ5508	A	No	0y1mo/F	Sep 2013	Cough, diarrhea, wheezing and respiratory failure	Severe pneumonia	RSV-B, PIV-3
CQ5571	B	Recurrent wheezing	4y2mo/F	Oct 2013	Cough, expectoration, and wheezing	Pneumonia, asthma	None
CQ5753	A	No	0y2mo/M	Nov 2013	Cough and rhinorrhea	Pneumonia	RSV-B
CQ7170	B	Asthma	2y9mo/M	Sep 2014	Cough, expectoration, wheezing and respiratory failure	Severe asthma, pneumonia	None
CQ7174	B	No	5y8mo/F	Sep 2014	Fever (38.2), cough, and wheezing	Moderate asthma, pneumonia	None
CQ7188	B	Recurrent wheezing	2y4mo/F	Sep 2014	Fever (39.2), cough, and wheezing	Moderate asthma, pneumonia	None
CQ7208	B	Asthma	6y11mo/M	Sep 2014	Cough, Chest pain and wheezing	Severe asthma, pneumonia	HBoV
CQ7214	B	No	2y8mo/F	Sep 2014	Fever (40), cough and expectoration	*Mycoplasma* pneumonia	*Mycoplasma pneumoniae*
CQ7225	B	Recurrent wheezing	4y0mo/F	Sep 2014	Cough, wheezing and respiratory failure	Severe asthma, pneumonia	*Moraxella catarrhalis*
CQ7226	B	Recurrent wheezing	5y11mo/M	Sep 2014	Cough, expectoration, chest pain, wheezing and respiratory failure	Severe asthma	None
CQ7233	B	Recurrent wheezing	1y11mo/F	Sep 2014	Cough, rhinorrhea and wheezing	Moderate asthma, pneumonia	None
CQ7280	B	Asthma	1y3mo/M	Oct 2014	Cough and wheezing	Moderate asthma, pneumonia	None
CQ7283	B	Asthma	1y4mo/M	Sep 2014	Fever (38.7), cough, expectoration and wheezing	Moderate asthma, pneumonia	RSV-A, *Moraxella catarrhalis*
CQ7307	B	No	3y7mo/M	Oct 2014	Cough, rhinorrhea and diarrhea	Bronchitis	PIV-4, *Enterobacter cloacae*
CQ7349	B	Asthma	3y11mo/M	Oct 2014	Cough and wheezing	Moderate asthma, pneumonia	RSV-B, *Streptococcus pneumoniae*
CQ7360	B	Asthma	10y8mo/F	Oct 2014	Cough, wheezing, respiratory failure and diarrhea	Severe asthma pneumonia	None

**Table 2:** Comparison of the clinical characteristics of the two clades of children with EV-D68.

Patient characteristic	EV-D68 N (%)				Clade N (%)		
	Total	Single detection	Co-detection	*P*value	Clade A	Clade B	*P*value
Number	19	8	11		4	15	
Gender (male)	10 (53)	3 (38)	7 (64)	0.370	2 (50)	8 (53)	1.00
Median age (months)	32 (1–128)	41.5 (16–128)	16 (1–83)	0.091	4.5 (1–7)	43 (16–128)	**0.001**
Median (days)							
Duration of hospitalization	6 (3–11)	4.5 (3–7)	6 (3–11)	0.152	8.5 (5–11)	5 (3–7)	**0.027**
Past history							
A past history of asthma	7 (37)	3 (38)	4 (36)	1.0	0	7 (47)	0.245
A past history of recurrent wheezing	13 (68)	7 (88)	6 (55)	0.177	1 (25)	12 (80)	0.071
Clinical symptoms							
Cough	19 (100)	8 (100)	11 (100)	/	4 (100)	15 (100)	/
Fever	5 (26)	2 (25)	3 (27)	1.0	1 (25)	4 (27)	1.0
Wheezing	17 (89)	8 (100)	9 (82)	0.485	4 (100)	13 (87)	1.0
Diagnosis							
A discharge diagnosis of asthma	13 (68)	8 (100)	5 (45)	**0.018**	0	13 (87)	**0.004**
Acute asthma exacerbations	11 (58)	7 (88)	4 (36)	0.059	0	11 (73)	**0.018**

**Table 3:** Pathogen spectrum of children hospitalized with acute asthma exacerbation.

Virus^a^	Number (Detection rate, %)			*P*^b^
	Total N = 75	September and October		
		2012 and 2013 N = 15	2014 N = 14	
EV-D68	11(15)	0	11(79)	<0.001
HRV	25(33)	7(47)	2(14)	0.11
RSV	17(23)	6(40)	5(36)	0.81
PIV	8(11)	1(7)	0	/
HBoV	8(11)	0	1(7)	/
IV	5(7)	1(7)	0	/
ADV	4(5)	1(7)	0	/
HMPV	2(3)	0	0	/

^a^EV-D68:enterovirus D68; HRV: human rhinovirus; RSV: respiratory syncytial virus; PIV: human parainfluenza viruses; HBoV: human bocavirus; IV: influenza viruses; ADV: adenovirus; HMPV: human metapneumovirus.

^b^The 2014 group compared with the 2012 and 2013 group.

**Table 4:** Primers used for detection and analysis of EV-D68.

Primer	Sequence (5′-3′)	Location (position^a^)
DK001^11^	CAAGCACTTCTGTTTCCC	5′UTR (164-168)
DK004^11^	CACGGACACCCAAAGTAGT	5′UTR (483-501)
Rhinoseq-FW^15^	GGGACCAACTACTTTGGGTGTCCGTGT	5′UTR (534-560)
Rhinoseq-RV^15^	GCATCIGGYARYTTCCACCACCANCC	VP2 (1168-1193)
VP4F^16^	GGACCCATCAAAATTCACTG	VP4(876-895)
VP2-2R^16^	CCATTGATGTGGAAATATTG	VP2(1451-1470)
VP2-F^16^	CCAGGGTTCGATGATATCATG	VP2 (1360-1380)
VP3-R^16^	GGCCCGTCTAACTGTATGTC	VP3 (1944-1963)
VP3-F^16^	GCACATTCCAGGGCAGGTCC	VP3 (1785-1804)
VP1RFH^16^	CACCAAGTTCGGGCGTTAATC	VP1 (2469-2488)
VP1F^16^	ACCATTTACATGCAGCAGAGG	VP1(2393-2413)
485^16^	ACATCTGAYTGCCA RTCYAC	2A (3425-3406)
EV68-1-F^b^	TGGGGTTGTTCCCACYCCAAA	5′UTR (13-33)
EV68-1-R^b^	ATCRRCCCAAGCTACACACG	5′UTR (303-322)
EV68-2-F^b^	TTGTTGAYGCGTTGCGCT	5′UTR (273-290)
EV68-2-R^b^	CCATTTGTRGCRATGTTGGC	5′UTR (779-798)
EV68-3-F^b^	AGAGCACCAAATGCVCTCAA	VP1 (3238-3257)
EV68-3-R^b^	GAGCATTGCATGCYTCHGTG	2C (4077-4096)
EV68-4-F^b^	GCCACRYTRGCATTGYTRGG	2B (3958-3977)
EV68-4-R^b^	GCCYTGAATRCCAGCAAAAA	3A (5294-5313)
EV68-5-F^b^	CCRGCTCCTGATGCYATAAA	3C (5472-5491)
EV68-5-R^b^	GTGHGTYGGTGTACCRCCTA	3D (6198-6217)
EV68-6-F^b^	AATGGAGCYCAAGGRTTTGC	3D (5869-5888)
EV68-6-R^b^	GTAGTGTCAAYGCCCTTCCC	3′UTR (7236-7255)

^a^Numbers correspond to the genome of EV-D68 Fermon strain (GenBank accession no. AY426531).

^b^Primers designed by Primer-BLAST. UTR, untranslated regions; VP, viral protein; F, forward; R, reverse.
