# Supplementary material for: Prevalence and molecular characterizations of enterovirus D68 among children with acute respiratory infection in China between 2012 and 2014
Source: Sci Rep. 2015 Nov 16;5:16639. doi: 10.1038/srep16639 (PMC4644992; doi:10.1038/srep16639)
Supplement: Supplementary Information [file srep16639-s1.pdf]

Prevalence and molecular characterizations of enterovirus D68 among children with acute respiratory infection in China between 2012 and 2014

Qiuyan Xiao[1], MD, Luo Ren[1], MD, PhD, Shouyan Zheng[1], MD, Lili Wang [1], MD, Xiaohong Xie[2], MD, PhD, Yu Deng[2], MD, PhD, Yao Zhao[1], MD, PhD, Xiaodong Zhao[1], MD, PhD, Zhengxiu Luo[2], MD, PhD, Zhou Fu[2], MD, PhD, Ailong Huang[3], MD, PhD, Enmei Liu[2] *, MD, PhD

Affiliations: [1] Ministry of Education Key Laboratory of Child Development and Disorders, Key Laboratory of Pediatrics in Chongqing, Chongqing International Science and Technology Cooperation Center for Child Development and Disorders, Chongqing, 400014, China. [2] Department of Respiratory Medicine, Children's Hospital of Chongqing Medical University, Chongqing, 400014, China. [3] Key Laboratory of Molecular Biology of Infectious Diseases, Ministry of Education, Chongqing Medical University, Chongqing, 400014, China

***Corresponding author：** Enmei Liu, Department of Respiratory Medicine, Children's Hospital of Chongqing Medical University, No. 136, the 2nd Zhongshan Road, Yuzhong District Chongqing, China, 400014, [emliu186@hotmail.com] Fax: +86-23-63622754, Tel: +86-23-63632386.

Figure legends

Figure S1. Analysis of complete VP1 amino acid sequences of EV-D68 among clade A, B and C.

The positions where the sequences of strains used for this analysis had amino acid residues identical to those of strain Fermon are indicated with dots. The BC and DE loop regions in VP1 are boxed.

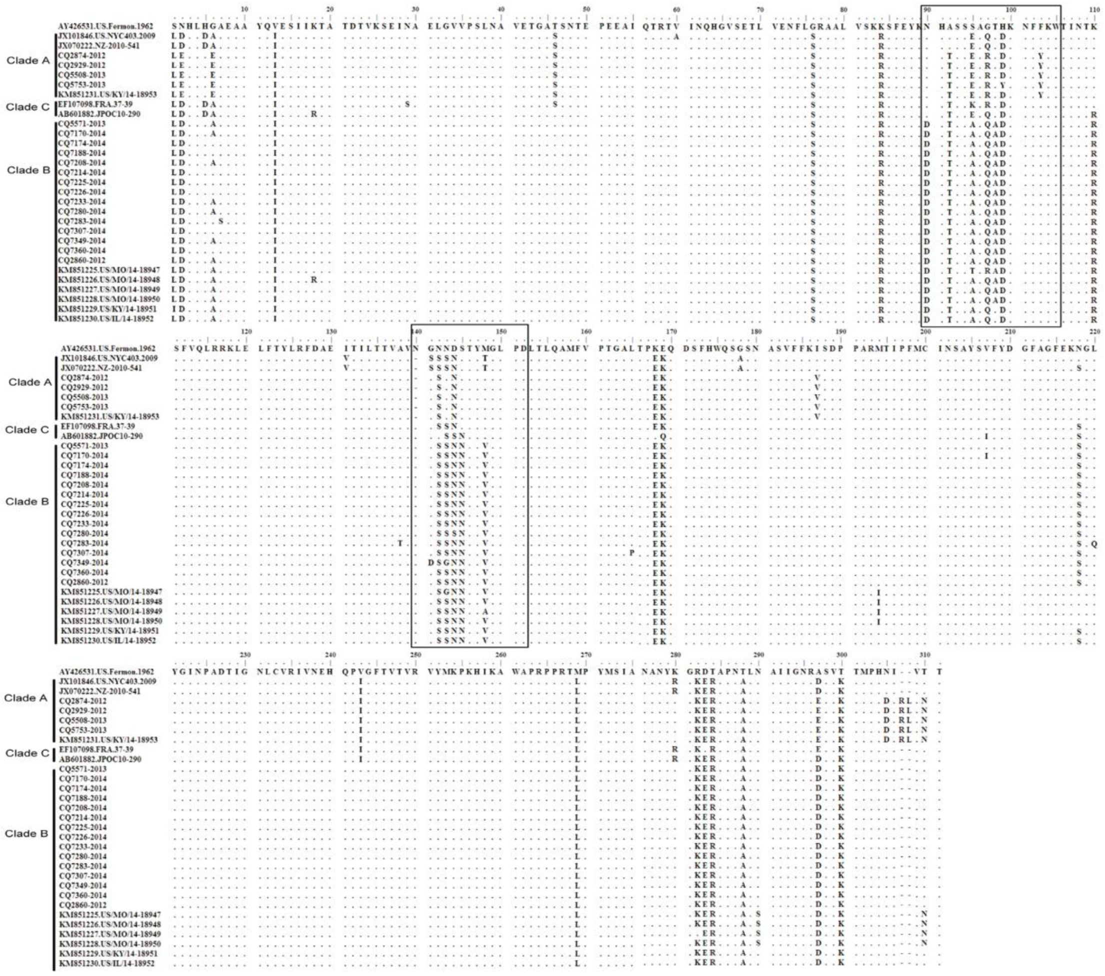

AY426531.US.Fermon.1962
Clade A
JX101846.US.NYC403.2009
JX070222.NZ-2010-541
CQ2874-2012
CQ2929-2012
CQ5508-2013
CQ5753-2013
KM851231.US/KY/14-18953
Clade C
EF107098.FRA.37-39
AB601882.JPOC10-290
Clade B
CQ5571-2013
CQ7170-2014
CQ7174-2014
CQ7188-2014
CQ7208-2014
CQ7214-2014
CQ7225-2014
CQ7226-2014
CQ7233-2014
CQ7280-2014
CQ7283-2014
CQ7307-2014
CQ7349-2014
CQ7360-2014
CQ2860-2012
KM851225.US/MO/14-18947
KM851226.US/MO/14-18948
KM851227.US/MO/14-18949
KM851228.US/MO/14-18950
KM851229.US/KY/14-18951
KM851230.US/IL/14-18952
